# A qualitative study on the voluntariness of counselling and testing for HIV amongst antenatal clinic attendees: do women have a choice?

**DOI:** 10.1186/s12910-018-0329-7

**Published:** 2018-11-21

**Authors:** Tausi S. Haruna, Evelyne Assenga, Judith Shayo

**Affiliations:** 1 Hubert Kairuki Memorial University, P.O.Box 65300, Dar es Salaam, Tanzania; 2 Muhimbili University of Health and Allied Sciences, P.O. Box 65001, Dar es salaam, Tanzania

**Keywords:** Provider-initiated-counselling and testing for HIV, Opt-out, Opt-in, HIV test amongst antenatal clinic attendees, Ethical dilemmas inherent in PITC, Risk of coercion with HIV test, Voluntary HIV test

## Abstract

**Background:**

Mother-to-child transmission (MTCT) of the Human Immunodeficiency –Virus (HIV) is a serious public health problem, contributing up to 90% of childhood HIV infections. In Tanzania, the prevention-of-mother-to-child-transmission (PMTCT) feature of the HIV programme was rolled out in 2000. The components of PMTCT include counselling and HIV testing directed at antenatal clinic attendees. It is through the process of Provider Initiated Counseling and Testing (PITC) that counselling is offered participant confidentiality and voluntariness are upheld and valid consent obtained.

The objective of the study was to explore antenatal clinic attendees’ experiences of the concept of voluntariness vis- a- vis the implementation of prior counseling and subsequent testing for HIV under the PITC as part of their antenatal care.

**Methods:**

In-depth interviews were conducted with17 antenatal clinic attendees and 6 nursing officers working at the Muhimbili National Hospital (MNH) antenatal clinic**.** The study data were analyzed using qualitative content analysis.

**Results:**

Antenatal clinic attendees’ accounts suggested that counselling and testing for HIV during pregnancy was voluntary, and that knowledge of their HIV status led them to access appropriate treatment for both mother and her newborn baby. They reported feeling no pressure from nursing officers, and gave verbal consent to undergo the HIV test. However, some antenatal clinic attendees reported pressure from their partners to test for HIV. Healthcare providers were thus faced with a dilemma of disclosure/ nondisclosure when dealing with discordant couples.

**Conclusion:**

Antenatal clinic attendees at MNH undertook the PITC for HIV voluntarily. This was enhanced by their prior knowledge of HIV, the need to prevent mother- to- child transmission of HIV, and the effectiveness of the voluntary policy implemented by nursing officers.

## Background

Mother-to-child transmission (MTCT) of HIV is a serious public health problem [1]. According to the World Health Organization (WHO), MTCT contributes up to 90% of childhood HIV infections. During 1998, in order to reduce vertical transmission of HIV, the WHO and key stakeholders recommended the initiation of a prevention-of-mother-to-child transmission programme (PMTCT) [2].

Voluntary counselling and testing for HIV (VCT) is a client-initiated model through which individuals can be made aware of their HIV status. This traditional approach of HIV testing and counselling was underpinned by individual’s rights to know his or her HIV status [3]. However, this approach has been shown to be inadequate. This is because it fails to capture key groups of people such as pregnant women who present themselves to Reproductive and Child Health (RCH) clinics for antenatal care and HIV-related services [4]. As a result, this approach did not bode well with the PMTCT programme, that had been started earlier in 2000 and had resulted in a low uptake of HIV testing. It was estimated that 1,194,172 women attending centers registered to provide PMTCT in 2009 [5], compared to 85% of pregnant women attending antenatal clinics were tested for HIV according to figures from the Tanzania Ministry of Health in the Global Aids Response Country Progress Report (2014). This significant increase has helped reduce the incidence of MTCT by 48% from 26,000 children in 2009 to 15,000 in 2012 [6].

Therefore, in order to increase the uptake of PMTCT services among pregnant women, in 2007 the WHO introduced a new approach for HIV testing known as Provider Initiated Counseling and Testing (PITC) [7]. It involves approaches such as Opt-in and Opt –out, the latter of the two having the most relevance for PMTCT services. In the opt-out approach women will be tested unless they actively decline after being offered pre-test information [3, 7]. In the Opt-in approach women give consent for the HIV test specifically [5]. Both approaches are ethically acceptable if women are made aware of their right to decline the test and are given opportunities to do so if that is their wish [5].

According to Beauchamp and Childress a person acts in a voluntary capacity when she /he has chosen to act with adequate knowledge, in the absence of both psychological compulsion and external constraints [8]. A cross-sectional study conducted in Northern Tanzania involving 500 pregnant women revealed that 41.7% of the women were willing to accept voluntary counselling and HIV testing (VCT) [9]. However, the study also identified factors that influenced their willingness to accept VCT for HIV, that is, high personal susceptibility to HIV, barriers related to confidentiality, their partners’ involvement, religious beliefs, and the availability of alternative infant feeding options. The Health Belief Model (HBM) conceptual framework suggests that voluntariness for HIV testing may be enhanced by knowledge about the perceived benefits of HIV testing, and seriousness of the disease, personal risks associated with acquisition of HIV and other factors such as knowledge about HIV and cues to relevant actions to take [9, 10]. Collectively, these concepts may help form part of an information package to enable pregnant women to make well informed decisions and promote the ethical acceptability of HIV testing in pregnant women as a public health intervention. In addition, antenatal counselling and testing for HIV provides the principal means through which healthcare providers meet safety targets by reducing the risk of MTCT. However, it has also been shown to cause unfavourable reactions from some women’s partners. These include abandonment of their spouses, physical violence and discrimination [9].

Further, HIV voluntary testing and counselling can confer benefits on long-term public health goals which concur with public health ethics and principles of humanity [11].Of direct relevance to this research study is the ethical acceptability of the PITC policy based on the principles of benefits to them other, the fetus and society as a whole. However, some studies have also demonstrated the drawbacks of PITC if it does not comply with set principles. The Opt-out approach under PITC requires the healthcare providers to suggest and subsequently to test, pregnant mothers for HIV as part of their standard antenatal care, unless they actively decline [7, 12].

The other three core aspects of voluntary HIV testing, whether client-initiated or provider-initiated, should include counselling, confidentiality and consent (‘3Cs’). A qualitative study conducted in the Ukraine focusing on the ‘3Cs’parameters revealed gaps between actual policies and practices associated with HIV testing These findings raise concerns about safeguarding pregnant women’s autonomy. [13].

Regarding HIV counseling and testing, there is a substantial body of literature about information- giving/ exchange and competence to make a choice but little on the voluntariness involved in testing, although there are concerns that women feel pressured to accept the HIV test [3, 14]. Therefore, the objective of this study was to explore antenatal clinic attendees’ experiences of the concept of voluntariness vis-à-vis the implementation of prior counselling and subsequent testing for HIV under the PITC as part of their antenatal care.

## Methods

### Design

An exploratory qualitative case study design was adopted to gain a better understanding about the voluntary nature of counselling and testing of HIV amongst antenatal clinic attendees at MNH. In-depth interviews were conducted with 17 antenatal attendees and 6 nursing officers working in the PITC section, using an interview guide comprised of open- ended questions.

### Study area

The study was conducted at the MNH antenatal clinic. This clinic was selected specifically because it serves the largest number of pregnant women from different social backgrounds in the Dar es Salaam region. It is also the national referral hospital in Tanzania, which provides RCH services including: antenatal care, out-patient and in-patient obstetric care, family planning services, PITC for pregnant women, and PMTCT of HIV for newborns of HIV positive mothers. On average, the RCH section cares for between 120 and 145 clients daily and between 2800 and 3000 clients monthly.

### Ethical considerations

Ethical approval to carry out this research study was obtained from Muhimbili University of Health and Allied Sciences (MUHAS) Research and Publication Committee (reference numbers; MU/PGS/SAEC/Vol.IX). Permission to conduct the research was requested from the MNH authorities. Separate written informed consent was obtained from antenatal clinic attendees and nursing officers respectively.

Participants also consented to having their interview audio- recorded and a study code number was assigned for both antenatal clinic attendees and nursing officers so as to ensure their anonymity.

### Participants: Antenatal attendees

The study participants were antenatal clinic attendees aged 18 years and above attending ANC services and healthcare providers engaged in routine PITC at MNH. The antenatal clinic attendees were enrolled using purposive sampling techniques. This approach was utilised to obtain a representative sample that included various criteria, namely - participants’ socio-demographic data, parity, and information relating to previous testing for HIV. Table 1 sets out the study participants’ socio-demographic characteristics.

**Table 1:** Study participants’ socio- demographic characteristics. Antenatal clinic attendees

Study participants’ characteristics.	Total 17 = *n* (%)
Age	
25–30	10 (58.8)
31–36	7 (41.2)
Marital status	
Married	12 (70.6)
Cohabiting/Engaged	3 (17.6)
Divorced/Separated	1 (5.9)
Single	1 (5.9)
Education level	
Primary education	3 (17.6)
Secondary education	5 (29.4)
Higher education	9 (53.0)
Employment status	
Employed	7 (41.2)
Unemployed	5 (29.4)
Self- employed	5 (29.4)
Parity	
Primigravid (not first visit)	6 (35.3)
Para 2–4	8 (47.1)
Para 5–6	3 (17.7)
Residential area	
Kinondoni	6 (35.9)
Ilala	3 (17.6)
Temeke	4 (23.5)
Ubungo	2 (11.8)
Kigamboni	2 (11.8)
Social support	
Spouse or family member	6 (35.3)
No spouse or family member	11(64.7)

### Participants: Nursing officers

The MNH antenatal clinic has nine nursing officers, that is, two working at the newborn vaccination unit and one who is in charge of the unit whose role is administrative. The remaining six nursing officers were routinely involved with counselling and testing for HIV and also provided PMTCT services for antenatal clinic attendees. Table 2 shows the socio-demographic characteristics of the nursing officers recruited in this study.

**Table 2:** Socio demographic characteristics Nursing Officers

Study participants’ characteristics.	Total 6 = *n* (%)
Level of education	
Certificate	1(16.7)
Diploma	4(66.7)
Advanced diploma	1(16.7)
Work experience	
<10 years	1(16.7)
>10 years	5(83.3)
Length of service as a PITC	
<5 years	3(50.0)
5–10 years	2(33.3)
>10 years	1(16.7)
Job title	
Senior assistant nursing officers	3 (50)
Senior nursing officer	1 (16.7)
Principal assistant nursing officer	1 (16.7)
Principal nursing officer	1 (16.7)
Gender	
All female	6 (100)

### Inclusion criteria

Antenatal clinic attendees aged 18 years and above, who had received PITC services based at the designated ANC, and who consented to their involvement in the study. Nursing officers who gave consent, and who were routinely involved in rendering PITC services.

### Exclusion criteria

First visit primi gravid antenatal clinic attendees if they had not been tested for HIV and hence might have underestimated the concepts under the study.

### Interview schedule/topic guide

In this research study the participants were asked a range of questions such as: “How did they perceive the PITC services? Whether they felt that they made a choice voluntarily, or whether they were forced to test for HIV. What behaviours impacted on their decision to accept the HIV test? Whether there were any restrictions that were unavoidable with the decision- making process. Furthermore, they were also asked about their understanding about HIV testing during pregnancy, its implications, reasons for the test, clinical and preventative benefits and the potential negative outcomes”. Nursing officers were requested to respond to a different set of questions, such as: “What were their perceptions of the differences between VCT and PITC? Whether they thought that antenatal attendees were free to accept or decline the HIV test, and what professional challenges they had encountered during the implementation of the PITC”.

### Data collection

In order to identify a relevant potential sample of antenatal clinic attendees for the in-depth interview (IDI), the principal investigator (PI) and registered nurses working in the clinic reviewed the information documented on the women’s antenatal attendance record cards to determine whether or not they met the study’s inclusion criteria. Following routine checks and tests, women were approached and invited to participate in the research study. After their consultation with the obstetrician, the women who had agreed to participate were asked to come to a private room for a face-to-face interview with the principal investigator. The interviews were conducted in Swahili, which is the national language spoken by majority of the targeted population. This enabled antenatal clinic attendees to comprehend and participate as fully as possible in the study. The interview guide had been pre-tested as part of a pilot initiative amongst other antenatal attendees not included in the current study. Interviews with the nursing officers were conducted at an agreed appointment time so as not to interfere with their work schedule. All interviews were audio- recorded with participants’ prior consent.

### Data transcription

To enable the principal investigator to make sense of the data, and also to ensure they were understandable to others, interviews were confined to 2 to 3 per day. The PI listened to the audio- recordings whilst awaiting the next interview. Discussion between the principal investigator and the research assistant took place at the end of the working day. Before starting with transcription, the audio recordings were uploaded and stored in the principal investigator’s computer and all files labelled confidentially. All interviews were transcribed verbatim through the use of an F4 programme and saved. F4 is a computer programme that allows the user to easily transfer audio files into a verbatim format.

In order to ensure accuracy data transcription was done by the PI and the research assistant together. Because all the interviews were conducted in Swahili, the PI and the research assistant translated the transcripts into English, and then checked for errors. Interview transcripts were read one by one as the study continued. The advantages of this approach were twofold. First, it was valuable to identify follow-up questions or probes for subsequent interviews, and second, to make a decision about whether data saturation had been reached.

### Data analysis

Preliminary data coding was done using NVivo 11. Coding refers to the breaking down of the study data into small units of words called codes [15]. The data were then analysed with the help of Computer- Assisted Qualitative Data Analysis Software (CAQDAS) called NVivo11 [12]. Qualitative content analysis was the method of choice. It is used to analyse qualitative data and interpret its meaning [15]. After preliminary coding, the data were re-examined to identify primary coding categories which were funnelled down so as to form sub-categories. The sub-categories were broken down further into similar or dissimilar units, so as to form the main categories.

Codes that were derived directly from the transcripts were used as a guideline when coding. As part of an ongoing process, we developed a code book where we documented identified coding categories and from there new codes were attached to the appropriate existing code as coding proceeded. The purpose of this process was to systematically group text data into fewer content-related categories that share the same meaning.

In first stage of (early) coding, 12 codes emerged from antenatal clinic attendees’ accounts. They were subsequently reduced to ten sub-categories. The data was carefully scrutinized so as to exclude overlaps. As a final stage in the process, the sub-categories were combined into five main categories as shown in Table 3.

**Table 3:** Main categories and sub- categories. Antenatal clinic attendees

Category	Sub-categories	Illustrative quotes from respondents
Antenatal attendees felt that the HIV test was voluntary	Made prior decision Exercising their autonomy to decline the test.	*“No there was no force, I decided on my own to go for a test”* [3] *“I don’t want to lie, I like to go for the HIV test often, even if I am not pregnant”* [11] *I would agree because of the advantages for me and the unborn child* [17].
Antenatal attendees felt no pressure to undergo a test	Absence of compulsions Absence of constraints and coercion	*I did not even ask myself because I wanted it done* [9] *I was just ready for anything* [25] *I can say it has its advantage because you get a healthy child* [17] *I wanted to know my status* [6]
Antenatal attendees experienced worry during the process of counseling	Fear of positive results Adverse effects of partners’ extra marital affairs	*I was scared, I was so afraid* [14] *I am ready but I was worried about my husband* [7] *I was worried because I had not taken the HIV test for about 6 months* [12]
Voluntariness was influenced by a number of factors	Accessibility to PMTCT services Knowledge about HIV Affairs with highly susceptible HIV positive individuals	*Having a partner who is unfaithful* *I am eager to know my status* [6] *When pregnant I think it is important to check your health status* [5] *In order to get a healthy baby it is a must to agree to test* [12]
Antenatal attendees were already informed about testing prior to their first clinic visit	HIV test was not unexpected	*I expected to take some tests including the HIV test and to receive counselling* [10] *I knew that I will be tested* [14] *Taking the HIV test first gives you information about your own health status, and second it protects the unborn child* [11].

A similar approach was used for the nursing officers’ accounts, whereby nine codes were a bridged to form six sub-categories, and these were further reduced to form two main categories, as shown in Table 4.

**Table 4:** Main categories and sub- categories. Nursing Officers

Category	Sub-categories	Illustrative quotes from respondents
The importance of non-directive counseling and adequate preparation	Need to provide appropriate information and, supportive care. The importance of listening to women and respecting their views in order to foster mutual trust	*To be honest, there was a positive response: “We have never been prepared like this before. It is good to feel adequately prepared”. Hence adequate preparation does enhance acceptance rates* [2]*.* *A large percentage of mothers agree, although there are few who disagree, but not totally. Sometimes they will ask questions and then give the issue more thought* [6].
PITC was seen as a good approach regardless of its ethical dilemmas	Enhanced uptake of HIV test Presence of clinical guideline Disclosure Truth telling	*I see PITC is good because we get many women here* [1]. *We have books for guidance purposes* [2] *The most difficult situation is when there is a couple a man and a woman, and when you get the results one is positive the other negative* [4]

## Results

In this section, the qualitative findings have been grouped into various categories, sub-categories and illustrated with women’s direct quotations. These are presented in Tables 3 and 4. All quotations were assigned a code number so as to ensure participants’ anonymity. Five main categories from the antenatal clinic attendees and two main categories for nursing officers had most relevance to the research study’s aims and objectives.

### Antenatal clinic attendees felt that the HIV test was voluntary

Antenatal attendees’ accounts suggested that testing for HIV was voluntary. Several reasons to support the analysis and interpretation of findings emerged. First, having some prior knowledge about the regulations that enhance gaining access to treatment. Second, having confidence about the ways in which the test results might impact on the health of the baby and third, an opportunity to confirm their own HIV status. Attendees for the most part reported that they were given a choice regarding whether or not to take the HIV test. However, for some attendees, the decision to be tested for HIV was already made before they attended the RCH clinic. Indeed, one antenatal clinic attendee remarked:

*“The health provider advises mothers to go for the HIV test, but my decision to be tested came from my heart. I know that for me the test is a must in order to be more confident, as I have never been tested before. Since I know I am pregnant, and I am married, I agreed to test from my heart. There was no external pressure.” (*Antenatal attendee No.7)

### Antenatal clinic attendees felt no pressure to undergo the test

Antenatal clinic attendees interviewed said that they did not experience pressure from healthcare providers to undergo the HIV test. However, a few reported feeling external pressure from their partners to test for HIV. This was because it was perceived as a way of knowing the couple’s HIV status. One participant stated:

*“…Because most men are difficult, he may tell you in conversation at home: ‘If you take the test, I will already be aware of my own health status, regardless’. But the results can be different. ‘I can be positive, while he is negative, or he can be positive, while I am negative’ ” (*Antenatal attendee. 12).

### Antenatal clinic attendees experienced worry during the process of counselling

Antenatal clinic attendees in this study experienced fear and worry during the process of counselling. These feelings were apparent even for those who undertook the HIV test frequently and were sure of their previous results. Antenatal attendees understood that being HIV negative was not a permanent status. This is because HIV is associated with exposure to behaviours which put individuals at risk. One participant noted:

*“…Because it is a different situation now (pregnancy) I was worried. I was having thoughts that if I have the disease what effect it would have on the child. Therefore I was worried, even when receiving the results, at first I was afraid.” (*Antenatal attendee.8)

### Voluntariness was influenced by a number of factors

Antenatal clinic attendees mentioned various factors which influenced their decision to accept the test for HIV during pregnancy. These included: (1) Access to antiretroviral drugs in order to prevent transmission from mother- to- child; (2) The health of the mothers themselves, which was seen as a preventive measure; (3) The effect of extramarital affairs (4) Gaining access to various nutritional programmes for their infants and (5) HIV knowledge and its implications for pregnant women. The following quotation summarizes one woman’s thoughts:

***“****First, I see that when you take the HIV test, you can be made aware of your health status. How can I protect myself, especially when I am negative? How can I advise my partner? Another thing is the protection of the baby, because when you are tested they can tell you about the various ways of passing on the virus, For example, it can be through breastfeeding, or through sores. So if I don’t want to transmit the disease I need to know the facts” (*Antenatal attendee 6)

Other factors included the influence of nursing officers, previous affairs with highly susceptible HIV positive individuals and the availability of social support, for example, being accompanied by their husband during antenatal visits. This is borne out in the following quotation:

*“First, having a partner who is unfaithful makes him interested in knowing if he is safe. The same can be said if his past sexual history indicates he had relations with someone suspected of having HIV. For example, if you are in a relationship with someone you don’t know, or with a person who is known to be HIV positive, testing has to be done.” (*Antenatal attendee 4)

*“… I felt relaxed because I trust myself, and I also trust my husband. We came together and got tested.”* (Antenatal attendee 13)

### Antenatal clinic attendees were already informed about HIV testing prior to their first clinic visit

Antenatal attendees were found to have accurate information about HIV and its implications for pregnant women. They reported that they received information from nursing officers based at the clinic during their routine antenatal care. For example, one remarked:

*“I was given advice about the test.: A mother should be aware of her health status, and also how to protect the baby. We were told that there are several ways to transmit HIV to the baby. In addition, if the mother is HIV positive, she is supposed to be kept on the correct dose of ART so as to protect the unborn child. The baby can also get HIV when still in the womb, during birth, and during breastfeeding*.*”* (Antenatal attendee 11)

Others reported receiving information by listening to the radio, and also through healthcare advertisements, social media, and during previous antenatal visits, as noted in the following quote:

*“Initially I heard it on the radio, then I heard a conversation while I was in the clinic: They said: ‘A pregnant woman nowadays can get a baby who is not affected”* (Antenatal attendee 17).

Antenatal clinic attendees had received post-test information which included information on the ‘window period’, preventive measures for HIV, advice on feeding options, and nursing officers’ recommendations for their partners to take the HIV test. The’ window period’ is defined as the time between HIV infection and seroconversion [7].Others reported the inadequacy of the post-test information at the time they received it

*“She gave the information to me before even showing me the results. She reminded me of the importance of taking the test. Then she gave me the results. She told me the results are for today and should be repeated after three months. She emphasized the importance of my spouse taking the test as well, so that we protect the baby and ourselves”* (Antenatal attendee 15).

Antenatal attendees reported that they trusted the nursing officers to maintain the confidentiality of the information shared between them and the healthcare team. A trusting relationship with healthcare providers helped provide a sense of security as illustrated in the following quotation:

*“I believe the information we shared was safe. There were no other people other than the healthcare provider and ourselves present when we had the test.”*(Antenatal attendee 5)

Other women, however, reported feeling a sense of lack of privacy during group counselling and this limited their opportunities to ask questions.

### Results: Nursing officers

#### The importance of non-directive counseling and adequate preparation

Nursing Officers’ responses suggested the importance of preparatory measures as a means of client support. These included counseling, guidance, respecting women’s views and, where appropriate, explanation and education. Collectively, these measures helped contribute to a relationship of mutual trust. One respondent said:

*“To be honest, there was a positive response. Some reported: “We have never been prepared like this before. It is good to feel adequately prepared”. Adequate preparations do enhance the acceptance rate”.* (Senior nursing officer III)

The importance of listening to women and respecting their views in order to foster mutual trust was reported by another nursing officer as illustrated in the following excerpt;

*“A large percentage of mothers agree, although there are few who disagree, but not totally. Sometimes they will ask questions and then give the issue more thought”.* (Senior assistant nursing officer)

#### PITC was seen as a good approach regardless of its ethical dilemmas

All six nursing officers interviewed perceived PITC to be a good approach when correctly implemented according to protocol. They noted that there were ethical dilemmas encountered when implementing the PITC, namely, confidentiality, disclosure, truth-telling and respecting the woman’s autonomy, as illustrated in the following quotation:

…“*Another challenge that confronts us is that a mother can come with her partner to be tested. They can take the test and await the results .When the results are available, one of them can refuse to listen to them, or she/he can quit, so it becomes difficult. For example, one day I tested a couple, but the man refused outright to receive post-test results and then quit. We tried to persuade him but he was totally unco-operative. What about the results? The mother was positive; the father who quit was negative, so it became difficult. Hence you start thinking: How am I going to help him? Then the mother starts asking: “What about his health status”? We have guidance and laws which inform us that the results are kept confidential for anyone who refuses to them. You can see the challenge”.***(**Senior nurse officer III)

## Discussion

Analysis and interpretation of the study’s findings have shown how the PITC services for HIV have impacted on pregnant women’s care. The study was conducted amongst antenatal clinic attendees of various social backgrounds, gestation, parity and residential areas. This presented the researchers with opportunities to explore different scenarios, thus contributing to the richness of the study data. The study revealed that antenatal attendees accepted the test without external pressure. Their decision was underpinned by prior knowledge about HIV and the advantages and disadvantages of testing during pregnancy. The antenatal clinic attendees were aware of the fact that pregnant women can transmit HIV to the unborn baby, a finding which concurs with other studies [14, 16].

The motivating factors associated with voluntariness towards PITC for HIV services amongst pregnant women included: access to Antiretroviral therapy (ART) drugs and infant feeding options, which were associated with having a baby who is not HIV-infected. This finding was consistent with other studies [10, 17]. In addition, other factors that facilitated voluntariness for the HIV test were: suspicion of extra-marital affairs, previous affairs with highly susceptible HIV positive individuals and the presence of spouse as a support person during the antenatal visits. However, even pregnant women who were confident about their husbands’ fidelity were equally ready to accept the HIV test.

It is possible that the availability of PITC for pregnant women at the clinic was viewed as a standard antenatal care procedure, and hence some antenatal attendees were unconcerned as to whether the test was voluntary or mandatory. They perceived it as a way of obtaining knowledge about HIV. Similar findings on the acceptability of PITC for HIV have been reported in Northwest Ethiopia [18]. This shows that if the guidelines of PITC for HIV are followed, it can help pregnant women consider their options in a supportive environment, cared for by healthcare providers whose approach is neutral.

The MoHSW guidelines for HIV Testing and Counselling in Clinical Setting (2007) recommend that counselling, confidentiality and consent remain an integral part of PITC and should be observed in accordance with good clinical practice [4]. The findings from this study showed that nursing officers recognised the importance of informed choice for antenatal attendees. A literature review on consent, counselling and confidentiality undertakenby the researchers showed the need to probe in greater depth the responses of antenatal mothers who do not know about the 3Cs. Additionally, lack of expertise by healthcare providers on the 3 Cs, may lead them to overlook one of the components during everyday clinical practice [13]. The responses of the antenatal clinic attendees in this study revealed that they gave verbal consent after understanding and balancing the benefits and risks of the HIV test during pregnancy. Their willingness to give consent may have been influenced by the fact that all six nursing officers interviewed were reported to have attended comprehensive training on PITC. However, on the downside, the nature of PITC does not provide antenatal clinic attendees with adequate time to understand the details of the given information because what they receive is just pre-test information that is characteristically not detailed [5]. Likewise, there is limited opportunity to go and think about the proposed offer.

The importance of non-directive counselling between women and nursing officers, was revealed in our study. Nursing officers’ experiences showed the importance of listening to, and respecting antenatal clinic attendees’views, in order to enhance mutual trust. This finding concurs with other studies which showed that a neutral counsellor who allows clients to make their own decisions is the preferred approach in prenatal counselling [20].

Furthermore, interviews with the nursing officers highlighted a number of ethical challenges when implementing PITC, namely: disclosure, confidentiality, transparency and respect for women’s autonomy. Disclosure of HIV status is the act of making HIV results known to a third person. In clinical practice, disclosure of test results has both negative and positive implications [21, 22].

According to the Tanzanian HIV and Acquired Immune Deficiency Syndrome (AIDS) guideline (2007), healthcare providers should only notify partners about positive results if they have given valid written consent [4]. Exceptions to this rule may occur where healthcare providers can disclose information to the respective partner after weighing into the balance the potential harm of nondisclosure in individual cases and after due consideration of appropriate measures to deal with the outcome. [19]. Some studies have associated disclosure of HIV status with negative outcomes, namely stigma, discrimination and violence against pregnant women. Other studies have shown that disclosure of HIV status to partners positively improved antiretroviral drugs adherence, and enhanced levels of social support [22–24].

Additionally, in this study, nursing officers also reported that giving results to discordant couples was ethically challenging because it created a risk of misunderstandings and/or marriage breakdown. Five of the nursing officers admitted that balancing their professional role and professional ethics was an ongoing challenge. However, couple testing was of great importance in treatment, care and support so as to improve both partners’ well- being. A study conducted in Zambia showed that couple counselling and testing had both medical and social benefits, but needed to be carefully planned and should not be under undue coercion [24].

### Future research

Our study has shown the importance of non-directive counseling and mutual trust between antenatal clinic attendees and nursing officers. Further studies could help determine whether non-directive counseling can be usefully integrated into other areas of clinical practice, such as antenatal screening for fetal abnormalities.

### Future practice

The PITC for HIV among antenatal clinic attendees was aimed at increasing the uptake of PMTCT services.Stakeholders should consider rolling out PITC more widely amongst other healthcare professionals.

### Study strengths

This study has provided an overview on the experiences of the antenatal attendees on the concept of voluntariness Categories were inductively synthesized with the support of sub-categories and illustrative quotations. The study also had minimal exclusion criteria: women recruited into the study had varied socio-economic backgrounds, educational qualifications, gestation and parity.

### Study limitations

The use of a purposely chosen small hospital-based sample of women may not have been wholly representative of the population of antenatal clinic attendees in Dar es Salaam. However, due to the fact that MNH provides healthcare services to women from the entire Dar es Salaam region, the study has given us an overview on the experiences of antenatal attendees on the concept of voluntariness on PITC for HIV within the region. Hence it is recommended that another study be done with a larger size study sample.

Conducting in-depth interviews at the antenatal clinic setting, where the participants were receiving their care, may also have influenced antenatal clinic attendees towards giving socially acceptable answers to the interview questions. The researchers tried to reduce this potential bias by asking open –ended questions, so that participants felt free to responding ways that were not socially desirable.

## Conclusion

Antenatal clinic attendees at MNH undertook the PITC for HIV voluntarily and the choice to opt-out was implicitly given. Most agreed to test for HIV. This was enhanced by their prior knowledge of HIV, the need to prevent mother- to- child transmission of HIV, and the effectiveness of the voluntary policy implementation by nursing officers. Antenatal clinic attendees were also aware that they need to receive pre-test information, have their privacy respected during PITC services, give their valid consent to be tested and also receive post-test information. Healthcare providers were faced with a dilemma of disclosure/nondisclosure when dealing with discordant couples.

### Recommendations

Nursing officers should continue to follow current guidelines and encourage antenatal clinic attendees who are undecided, or who are worried about HIV testing to consider their options carefully. This will also help minimize the fear of negative socio-cultural consequences of testing positive for HIV. Stakeholders should review the ethical aspects of disclosure guidelines pertaining to discordant couples so as to better facilitate and support the healthcare providers’work in this sensitive area of clinical practice.

## Abbreviations

3 C’s: Counselling, Confidentiality and Consent
AIDS: Acquired Immune Deficiency Syndrome
ANC: Antenatal Clinic
ART: Antiretroviral therapy
DMRET: Dartmouth/MUHAS Research Ethics Training
HIV: Human Immunodeficiency Virus
IDI: In-depth Interviews
MoHCDGEC: Ministry of Health, Community Development, Gender, Elderly, and Children
MoHSW: Ministry of Health and Social Welfare
MTCT: Mother -To -Child Transmission
NACP: National Aids Control Programme
PITC: Provider Initiated Testing and Counselling
PMTCT: Prevention of Mother- To- Child Transmission
RCH: Reproductive and Child Health
UNAIDS: United Nations Programme on HIV/AIDS
VCT: Voluntary Counselling and Testing
WHO: World Health Organization
